# Reducing toxic reactive carbonyl species in e-cigarette emissions: testing a harm-reduction strategy based on dicarbonyl trapping

**DOI:** 10.1039/d0ra02138e

**Published:** 2020-06-05

**Authors:** Bruna de Falco, Antonios Petridis, Poornima Paramasivan, Antonio Dario Troise, Andrea Scaloni, Yusuf Deeni, W. Edryd Stephens, Alberto Fiore

**Affiliations:** Division of Engineering and Food Science, School of Applied Science, University of Abertay Bell Street Dundee DD1 1HG UK A.Fiore@abertay.ac.uk +44 (0) 1382 308043; Division of Health Sciences, School of Applied Science, University of Abertay Bell Street Dundee DD1 1HG UK; School of Earth & Environmental Sciences, University of St Andrews Irvine Building, North Street, St Andrews, Fife KY16 9AL UK wes@st-andrews.ac.uk +44 (0) 1334 463947; Department of Agricultural Sciences, University of Naples II Portici 80055 Italy; Proteomics & Mass Spectrometry Laboratory, ISPAAM, National Research Council 80147 Naples Italy; Centre for Analytical Bioscience, Advanced Materials and Healthcare Technology Division, School of Pharmacy, University of Nottingham Nottingham NG7 2RD UK

## Abstract

Reducing the concentration of reactive carbonyl species (RCS) in e-cigarette emissions represents a major goal to control their potentially harmful effects. Here, we adopted a novel strategy of trapping carbonyls present in e-cigarette emissions by adding polyphenols in e-liquid formulations. Our work showed that the addition of gallic acid, hydroxytyrosol and epigallocatechin gallate reduced the levels of carbonyls formed in the aerosols of vaped e-cigarettes, including formaldehyde, methylglyoxal and glyoxal. Liquid chromatography mass spectrometry analysis highlighted the formation of covalent adducts between aromatic rings and dicarbonyls in both e-liquids and vaped samples, suggesting that dicarbonyls were formed in the e-liquids as degradation products of propylene glycol and glycerol before vaping. Short-term cytotoxic analysis on two lung cellular models showed that dicarbonyl-polyphenol adducts are not cytotoxic, even though carbonyl trapping did not improve cell viability. Our work sheds lights on the ability of polyphenols to trap RCS in high carbonyl e-cigarette emissions, suggesting their potential value in commercial e-liquid formulations.

## Abstract 2

Reducing the concentration of reactive carbonyl species (RCS) in e-cigarette emissions represents a major goal to control their potentially harmful effects.

## Graphical abstract

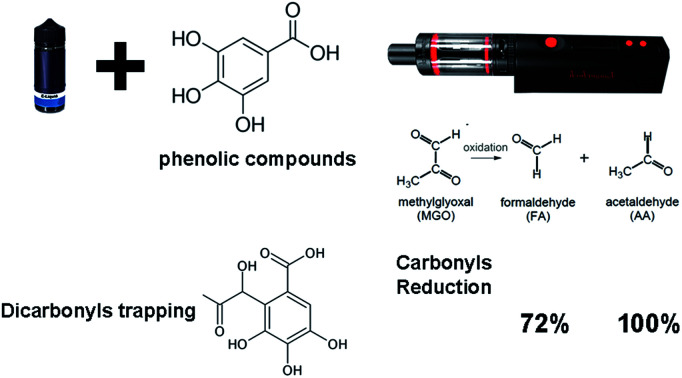

## Introduction

The use of e-cigarettes is a major issue in public health. Proponents of e-cigarettes stress the benefits to smokers during attempts to quit, while opponents fear that e-cigarettes will attract young people into vaping, thus developing an addiction to nicotine and possibly transition to combustible tobacco with time. All agree that e-cigarettes are harmful, but most agree that their emissions are less harmful than combustible cigarettes as they contain fewer carcinogens and toxicants, mostly in lower concentrations.^1,2^

E-cigarettes have been widely used for about a decade but given a latency period of two or more decades for many smoking-related symptoms and conditions to become manifest, it is too early to evaluate the long-term clinical effects of vaping on public health. In the meantime, approaches to predicting long term effects have been based mainly on extrapolating short-term clinical trials, chemical studies of emissions, and *in vitro* toxicology. Each of these indicates potentially toxic effects from e-cigarette emissions but generally at much lower levels when compared to cigarette smoke at equivalent levels of exposure.^3^

The WHO estimates that about half of all lifetime smokers will die of a smoking-related disease and 91% of these deaths will be directly attributable to cancer, cardiovascular or respiratory disease.^4^ Reactive carbonyl species (RCS) in the form of small aldehydes and ketones are biochemical contributors to each of these diseases. Formaldehyde and acetaldehyde are among the most potent carcinogens in tobacco smoke,^5^ acrolein is implicated in cardiovascular disease, while formaldehyde and acetaldehyde have respiratory effects.^6,7^ Some or all of these carbonyls can also be present in e-cigarette emissions in quantities that give rise to health concerns;^8^ it is clear that reducing their concentrations in emissions could represent a substantial contribution to harm reduction.

RCS are mainly produced by thermal degradation of glycerol and propylene glycol present in different ratios in the e-liquid, serving as carrier solvents. The coil temperature of e-cigarette devices may induce thermal decomposition of carrier solvents in highly reactive radicals, leading to low molecular weight carbonyl compounds such as formaldehyde, acetaldehyde, acrolein, acetone, methylglyoxal and glyoxal, all with established toxic effects on human health.^9–11^

Currently, most strategies for reducing toxicants found in e-cigarette emissions are based on the design of the product, *e.g.* by limiting the opportunity for metal contamination or preventing coils from reaching high temperatures that activate the degradation of e-liquid.^12,13^ However, only a few addressed the chemical nature of the liquid, most notably by using the propylene glycol/glycerol ratio to limit carbonyl production. A novel strategy of interrupting the oxidation and fragmentation pathways of propylene glycol and inhibiting the formation of RCS can include the use of trapping agents in the form of polyphenols to minimize the toxic concentrations of RCS in e-cigarette emissions.

Polyphenols occur naturally in many plants and food products^14–16^ and are considered to provide health benefits when ingested due to their *in vivo* antioxidant properties.^17^ The ability of phenolic compounds to trap RCS produced in different biological environments has been demonstrated.^18–21^ Besides their chelating and free radical scavenger functions, polyphenols can act as lipid- and carbohydrate-derived carbonyl scavengers.^22^ Sang and co-workers^23^ showed how the A-ring of epigallocatechin gallate can efficiently react with methylglyoxal to form mono and di-methylglyoxal adducts. Based on the chemical nature of polyphenols, the trapping reactions occur between the electrophilic carbon of either aldehydes or ketones and the catechol group *via* an electrophilic aromatic substitution. The presence of hydroxyl substituents on the aromatic ring affects both the regioselectivity and the speed of this reaction, activating and promoting the substitution in *ortho*/*para* positions.^24^

In this study, we exploited the biological and *in vitro* RCS trapping property of gallic acid, hydroxytyrosol and epigallocatechin gallate based on their chemical structures and in particular to their ability to form adducts with glyoxal and methylglyoxal.^19,23,24^ Moreover, we investigated the effects in a non-biological and *ex vivo* setting to trap glycerol and propylene glycol degradation products in the form of carbonyls and dicarbonyls present in the aerosols of vaped ECs.

## Material and methods

### Reagents and materials

Glycerol and propylene glycol, 2,4-dinitrophenylhydrazine (DNPH; 4% in phosphoric acid solution), *o*-phenylenediamine (*o*-PD), 2,4-dinitrophenylhydrazones of aldehyde/ketone–DNPH stock standard-13 (acetaldehyde–DNPH, acetone–DNPH, acrolein–DNPH, benzaldehyde–DNPH, 2-butanone–DNPH, *n*-butyraldehyde–DNPH, crotonaldehyde–DNPH, formaldehyde–DNPH, hexaldehyde–DNPH, methacrolein–DNPH, propionaldehyde–DNPH, *m*-tolualdehyde–DNPH, valeraldehyde–DNPH), methylglyoxal (40% aqueous solution), glyoxal (40% aqueous solution) and pure polyphenol standard, gallic acid, hydroxytyrosol and epigallocatechin gallate were obtained from Sigma-Aldrich (St. Louis, MO). Trizma base (Tris-(hydroxymethyl)-aminomethane ACS Reagent Grade), solvents for chromatography analysis, such as acetonitrile, methanol and water, (liquid chromatography-mass spectrometry, LC-MS grade), as well as acetic acid were purchased from Fisher Scientific (Loughborough, UK). Disodium hydrogen phosphate and sodium dihydrogen phosphate were purchased from Merck (Darmstadt, Germany). Fibrous 4 μm silica (“silica wool”) was obtained from H. Baumbach & Co Ltd, (Suffolk, UK) and Whatman 47 mm QMA silica filters were obtained from Sigma-Aldrich.

### Sample preparation

The e-liquid formulation was prepared, according to Stephens *et al.*,^25^ by gravimetrically weighing propylene glycol, glycerol and water in a ratio 70 : 20 : 10 (w/w/w) and used as the model e-liquid system. This e-liquid model system was used as a control sample and compared to e-liquid formulations in which water was replaced by standard polyphenol solutions. Gallic acid, hydroxytyrosol and epigallocatechin gallate were prepared at four concentrations (0.6; 1.25; 2.5 and 5 mM) in Milli-Q water. Each e-liquid formulation was then vortexed for 1 min, sonicated for 3 min to remove bubbles of air and stored at 4 °C until further use. The choice of the selected concentration of phenols was based on the following criteria: (i) previous data literature, (ii) cytotoxicity of these phenols on different cell lines, (iii) their toxicological data such as TDLo, LDLo, LD_50_ (Lowest Toxic Dose, Lowest Lethal Dose and Lethal Dose 50) and (iv) the amount of e-liquid that vapors inhale per day.^26–35^

### Laboratory vaping and aerosol collection

The Subox Mini C device (KangerTech, Shenzhen, China) was used as a recent generation vaping device in all experiments. This device incorporates circuitry capable of generating much higher output voltages to the atomiser than the nominal 3.7 V of the battery (a regulated box mod in vaping jargon). Another advantage is that the atomising coil can be easily removed for visual inspection between vaping runs, without disturbing the tank containing the e-liquid. The pump, solenoids and atomiser power supply and controller are part of the Gram Universal Vaping Machine package (UVM, Gram Health Inc., USA). The coil received an adequate supply of e-liquid throughout the run by creating holes in the cotton wrapping around the coil, which was found to reduce the incidence of wick deposits or burning.^25^ Aerosol collection and run conditions were carried out according to Stephens *et al.*^25^ Briefly, the aerosol was trapped in a plug of amorphous silica fibres (0.75 g of 4 μm diameter) within a 10 mL syringe inserted between the e-cigarette mouthpiece and the pump of the vaping machine. The atomiser was vaped with a power of 30 W to create high temperatures in the 1.5 ohm coil, which should lead to excess carbonyl production. A thin K-type thermocouple aligned along the axis of the coil indicated that temperatures of 166.4 °C (*s* = 4.5, *n* = 5) were achieved in the model e-liquid under these conditions. Twenty-five puffs of 55 mL were drawn over 4 s and repeated every 30 s. Condensate collection from the silica plug was achieved by centrifuging the 10 mL syringe within a 50 mL centrifuge tube at 4700 rpm, for 5 min. The condensate was immediately sealed and stored at −20 °C until required. In order to minimize contamination, 5 coils have been used to run experiments, one for all control samples and the others for gallic acid, hydroxytyrosol, and epigallocatechin gallate samples.

### HPLC-UV analysis of carbonyl compounds

Carbonyls quantification was carried out according to the industry-standard method (CORESTA method 74) with some modifications.^36^ Both e-liquid and condensed vapour were diluted in acetonitrile (1 : 10), and 50 μL of each sample was derivatised with 10 μL of 0.02 M DNPH for 25 min to allow the formation of DNPH-adducts. Samples were stabilized with Trizma® base solution (acetonitrile/aqueous Trizma 80 : 20) and analysed using high-performance liquid chromatography (HPLC) coupled to a UV detector. Chromatographic separations for both carbonyls and dicarbonyls were achieved using a Raptor ARC-18 (150 × 4.6 mm, 2.7 μm; Thames Restek, Saunderton, UK) column set at 40 °C. The HPLC-DAD system consisted of a Thermo Scientific Dionex UltiMate 3000 system (Fisher Scientific), composed of a degassing device, an ASI-100 automated sample injector and a PDA-100 photodiode array detector set at 365 nm. Separation of carbonyls was achieved by injecting 5 μL of derivatised sample using a flow rate of 0.6 mL min^−1^ and an elution gradient made of ultra-high purity water (solvent A) and acetonitrile mixed with methanol (1 : 14 v/v) (solvent B): 0 min, 70% B; 10 min, 75% B; 16 min, 90% B; 16.01 min, 100% B; 17 min, 100% B. Limits of quantification for formaldehyde and acetaldehyde were 0.171 and 0.135 μg mL^−1^, respectively, while corresponding limits of detection were 0.051 and 0.040 μg mL^−1^, respectively, in accordance to Stephens *et al.*, 2019.^25^

### HPLC-UV analysis of dicarbonyl compounds

Dicarbonyls were identified and quantified as described by Hellwig *et al.*,^37^ with the following modifications: 100 μL of diluted sample (1 : 10) was mixed with 30 μL of 0.2% *o*-PD in 9.6 mM EDTA solution and 30 μL of phosphate buffer solution (0.4 M, pH 7.0) in order to derivatise methylglyoxal and glyoxal into 2-methylquinoxaline (2-MQx) and quinoxaline (Qx), respectively. Samples were then incubated at 37 °C for 3 h and analysed. Dicarbonyls separation was achieved on a Raptor ARC-18 (150 × 4.6 mm, 2.7 μm; Thames Restek, Saunderton, UK) column by injecting 20 μL of derivatised sample, which was eluted with a gradient made of 0.075% acetic acid (solvent A) and acetonitrile (solvent B): 5 min, 2% B; 22 min, 70% B; 25 min, 70% B at a flow rate of 0.8 mL min^−1^. Compounds were identified by comparison with pure quinoxalines reference standards. Calibration curves for dicarbonyls were prepared in the range 0.06–3.00 μg mL^−1^ in acetonitrile. Limits of quantification and limits of detection for methylglyoxal and glyoxal were 0.03 and 0.01 μg mL^−1^, respectively. Results were expressed as μg mL^−1^.

### Liquid chromatography tandem mass spectrometry (HPLC-ESI-MS/MS) analysis

HPLC-ESI-MS/MS was carried out using a Nexera X2 system coupled to a LCMS 8040 triple-quadrupole mass spectrometer (Shimadzu Europa GmbH, Duisburg, Germany) according to the method described by Navarro and Morales.^38^ Samples were separated on a Raptor ARC-18 column (150 × 4.6 mm, 2.7 μm; Thames Restek Ltd, Saunderton, UK), at a constant temperature of 40 °C. The column was eluted with a gradient made of 0.5% acetic acid (solvent A) and methanol (solvent B): 0.1 min, 2% B; 1.5 min, 60% B; 31.5 min, 98% B; 5 min, 98% B, at a flow rate of 0.3 mL min^−1^. A 5 μL aliquot of each sample or standard compounds was injected for each run and elution profiles were detected using multiple reaction monitoring (MRM). For MS detection, ionization was carried out in negative mode and MS settings were optimized through direct flow-infusion of samples using the method optimisation function of the LabSolutions software (Shimadzu Europa GmbH, Duisburg, Germany). The automatic procedure included the following steps: scanning of total ion current; optimization and adjustment of precursor ions; auto-selection of product ions with consequent optimisation of collision energy (CE) with increments of 5 V; accurate optimisation of Q1 and Q3 voltages. MRM parameters were checked in the real run. The ion source settings were as follows: nebulizing gas flow: 3 L min^−1^; desolvation line (DL) temperature: 250 °C; heat block temperature: 400 °C; drying gas flow: 15 L min^−1^; collision-induced dissociation (CID) gas: 17 kPa; CE: 5–35 V. Data acquisition and analysis was performed using the LabSolutions software.

### Cell culture and cytotoxicity

The human lung adenocarcinoma cells A549 were maintained in DMEM (cat. no. 21885108, Gibco™, Fisher Scientific Ltd, UK) and normal human bronchial epithelial cells BEAS-2B were maintained in LHC-9 medium (cat. no. 12680013) supplemented with 10% FBS and 100 U/100 μg mL^−1^ of Pen/Strep (Gibco™, Fisher Scientific Ltd, UK), under a 5% carbon dioxide atmosphere, at 37 °C. Cell viability assay was performed as previously described^39,40^ but using CellTiter-glo3D (cat. no. G9682, Promega, UK) following manufacturers' instructions. Briefly, A549 and BEAS-2B cells were seeded at a density of 1 × 10^4^ in an opaque 96 well plate, and allowed to form monolayers, overnight. Then, media containing varying concentrations (final concentration ranging from 10.0–0.001 μM) of antioxidants with or without condensed aerosol were added, and incubated for 24 and 48 h. Control cells received water or antioxidants dissolved in water. After treatment, equal volume of media and CellTiter-glo3D were added, placed on a shaker for 2 min, and then equilibrated at room temperature for 10 min to stabilize the luminescence signal. Further, luminescence was measured using a Luminometer (Anthos Lucy1); the corresponding signal, which is directly proportional to the ATP level in viable cells, was used to calculate cell viability relative to control cells (water). The results were obtained in triplicates and expressed as mean ± SEM of viable cells relative to the control.

### Statistical analysis

All samples were analysed in three independent replicates and results are presented as mean values ± standard deviation of analytical and technical replicates. Data were analysed by ANOVA using XLStat (version 2014.5.03, Addinsoft, NY). Significant differences between samples with a confidence interval of 95% were determined using Tukey's range test.

## Results and discussion

### Reducing reactive carbonyl species

**Fig. 1:**
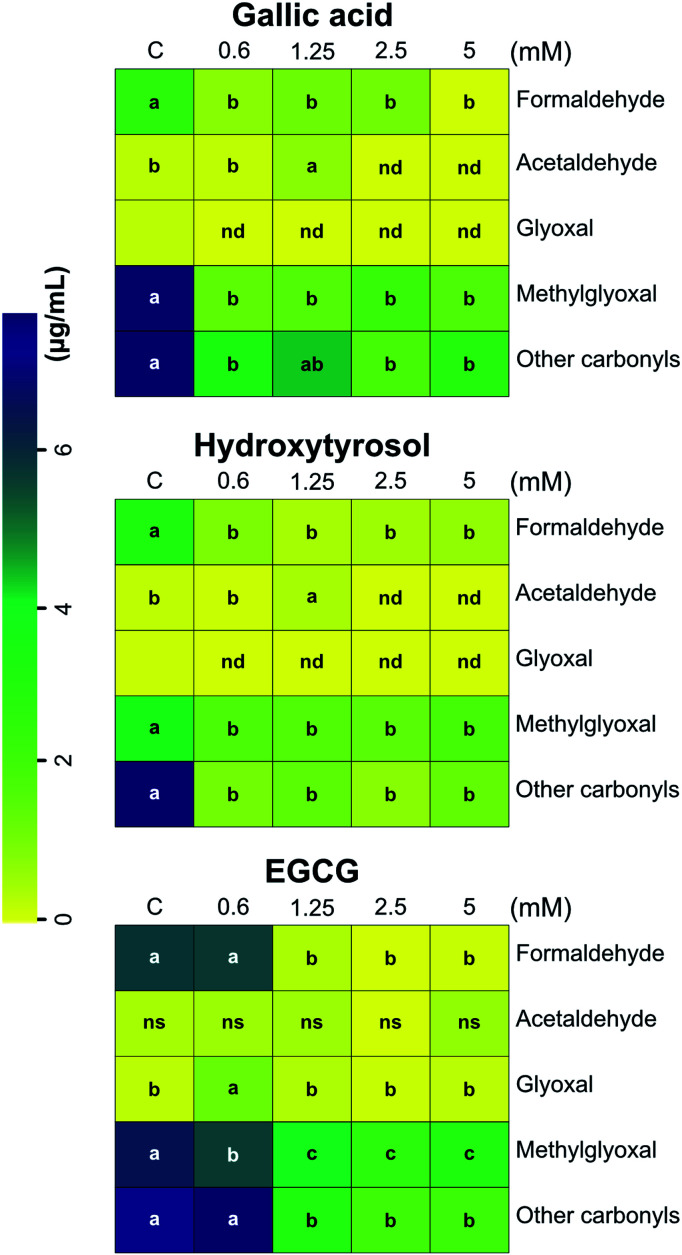
Heat-map showing the concentration of carbonyls and dicarbonyls in the aerosols of vaped e-cigarettes after addition of phenolic compounds in the e-liquid formulations. The addition of polyphenols reduced carbonyl and dicarbonyl concentration in the corresponding aerosols. Data were subjected to one-way ANOVA and significant differences (*P* ≤ 0.05) between means were determined using Tukey's test. Lowercase letters denote differences between treatments for each reactive carbonyl species. Each block is the mean value of three replicates ± SE, *n* = 3. C, control (model e-liquid system); EGCG, epigallocatechin gallate; nd, not detected; ns, not significant. Other carbonyls were area summed from known and unknown peaks as previously reported by Stephens *et al.*, 2019.^25^ Boxes without letters, below limit of quantification.

Several studies have demonstrated the capacity of polyphenols to trap RCS^23,24,41–43^ in solid model systems or in closed reactors. Three phenolic compounds (gallic acid, hydroxytyrosol, and epigallocatechin gallate) were used to reduce the levels of glycerol and propylene glycol degradation products present in the aerosols of vaped laboratory-formulated e-cigarettes, such as carbonyls and dicarbonyls. The addition of polyphenols in the e-liquid formulations reduced the concentration of RCS in the aerosol condensate, except for: (i) glyoxal in the experiment with gallic acid; (ii) acetaldehyde and glyoxal in the experiment with hydroxytyrosol; (iii) acetaldehyde and glyoxal in the experiment with epigallocatechin gallate. Moreover, the addition of 0.6 mM epigallocatechin gallate in the e-liquid formulation did not alter the concentration of carbonyls in the aerosol condensate, except for methylglyoxal (MGO) that was reduced by 14% (Fig. 1). In the gallic acid e-cigarette model system (0.6; 1.25; 2.5 and 5 mM), the concentration of formaldehyde, acetaldehyde, methylglyoxal, and other carbonyls decreased up to 99.6%, 100%, 82.2, and 76%, respectively, compared with the control model system. A similar trend was observed for hydroxytyrosol (formaldehyde, methylglyoxal and other carbonyls) and epigallocatechin gallate (formaldehyde, methylglyoxal, and other carbonyls) model systems. The concentrations of carbonyl compounds were in the same order of magnitude as those reported by Uchiyama *et al.*,^44^ which ranged from 0.3 to 61 μg mL^−1^, depending on the carbonyl compound and the e-cigarette brand. Formaldehyde and acetaldehyde were the main carbonyls identified and quantified in the condensed aerosols as DNPH derivatives. Specifically, we used DNPH because it is highly selective for carbonyl compounds, even in the presence of thiol groups and other nucleophiles in the sample, other DNPH azo-derivatives can be formed as well.^45^ In contrast to commercial e-liquids, which are rich in esters, alcohols, ketones and aldehydes with no sulphur present in their molecular structures,^46^ we used a laboratory-prepared model e-liquid system with no flavours or nicotine added and vaped at high power (30 W). This strategy maximised the generation of carbonyls and minimised the impact of added ingredients on the estimation of the unknown peaks, excluding them as putative contributors to the total amount of carbonyls formed.

### Trapping of carbonyls and formation of adducts

**Fig. 2:**
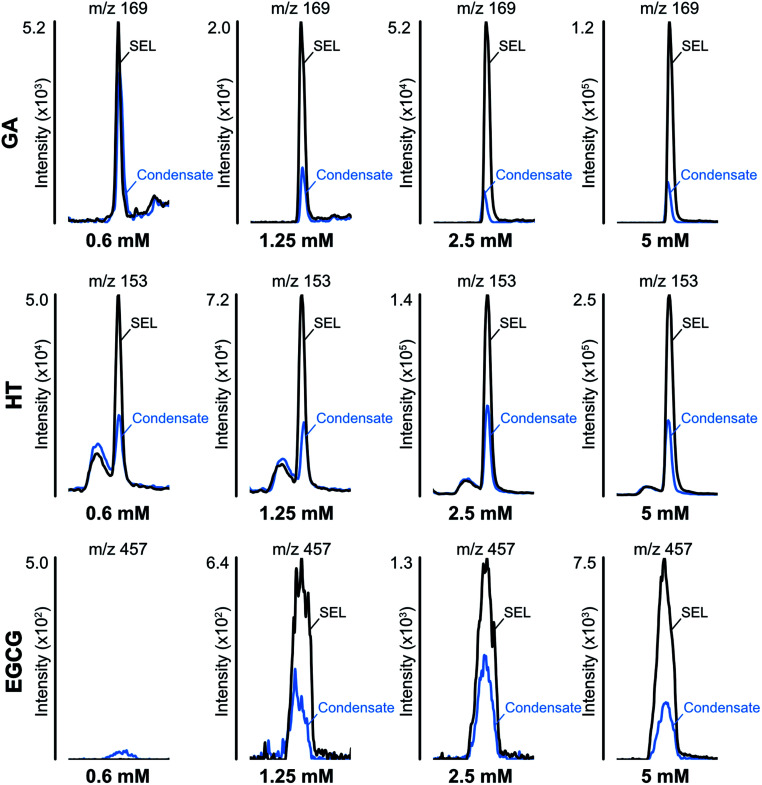
The reduction of polyphenols in the e-liquid (SEL) and aerosol condensate samples. GA, gallic acid; HT, hydroxytyrosol, EGCG, epigallocatechin gallate.

Fig. 2 shows the reduction of phenolic compounds in the e-liquid and aerosol condensate samples. The results indicate that the concentration of all polyphenols decreased after vaping, regardless of their initial concentration in the e-liquid (Fig. 2). The decrease of polyphenols ranged from 60–98%, implying their potential involvement in dicarbonyl trapping, as well as their oxidation and further reactions as condensation, polymerization or cleavage.^47^ These quantitative measurements took into account the compound recovery factors associated with e-liquid mixture volatilization (between 50 and 70 °C), and adsorption/release of phenols from silica plugs, as already determined by our group.^25^

**Fig. 3:**
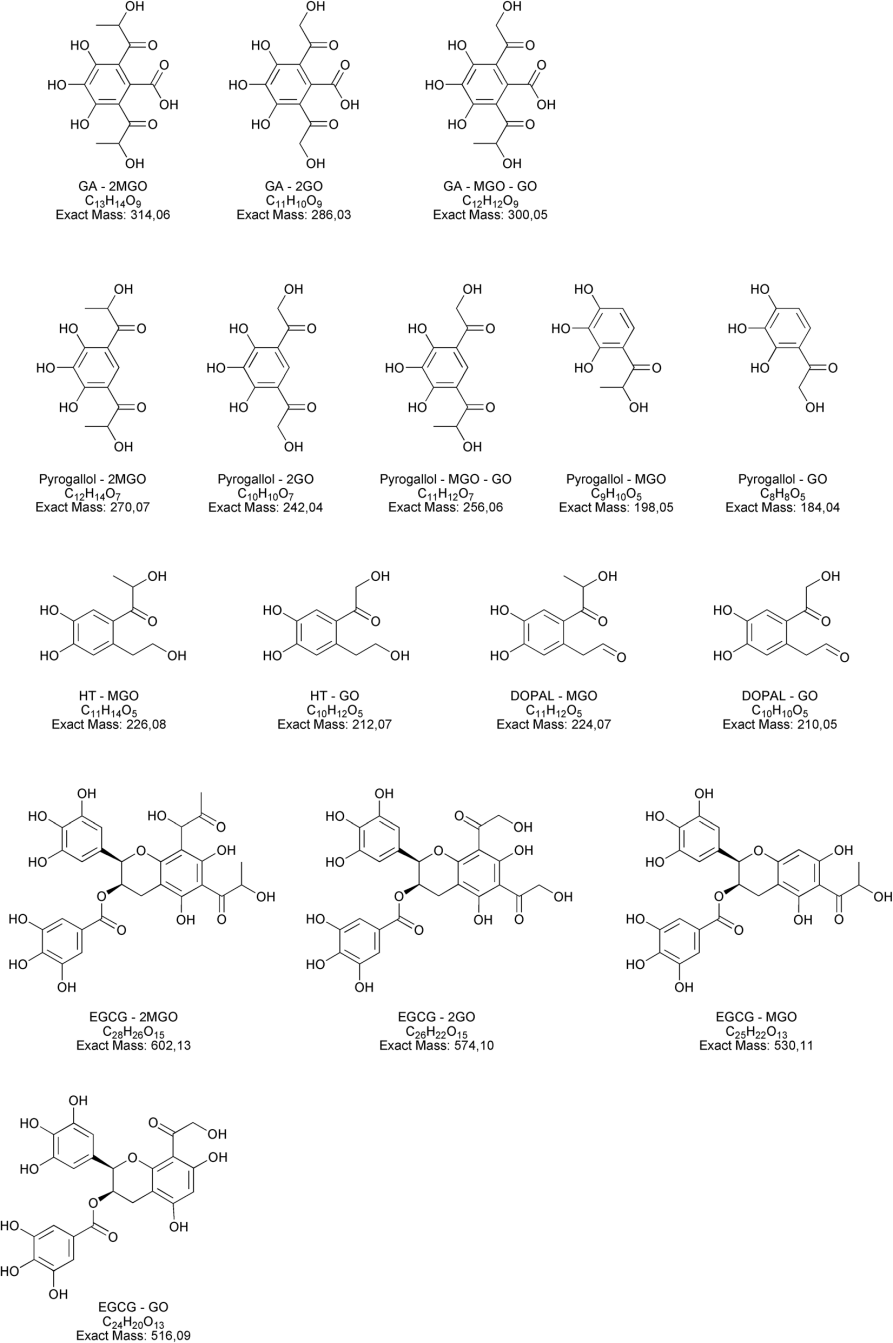
Molecular structures and their exact masses of hypothesized adducts. Abbreviations used: HT, hydroxytyrosol, EGCG, epigallocatechin gallate, DOPAL, 3,4-dihydroxyphenylacetaldehyde, GO, glyoxal, MGO, methylglyoxal.

**Fig. 4:**
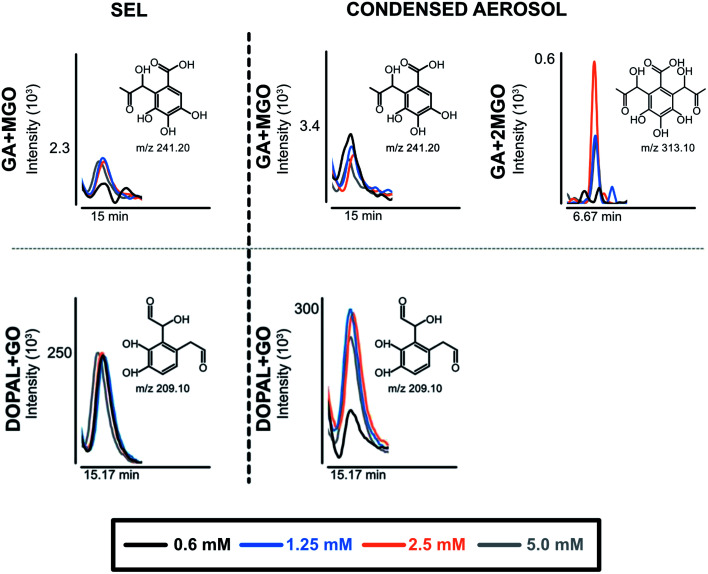
The formation of adducts between phenolic compounds and dicarbonyls in e-liquid (SEL) and aerosol condensate samples after addition of phenolic compounds in the e-liquid formulations. DOPAL, 3,4-dihydrophenyl-acetaldehyde; EGCG, epigallocatechin gallate; GA, gallic acid; GO, glyoxal; MGO, methylglyoxal.

Along with the reduction of dicarbonyls and polyphenols, we further investigated the formation of adducts by using tandem mass spectrometry. We focused on the formation of adducts between polyphenols and glyoxal or methylglyoxal or polyphenols derivatives as 3,4-dihydroxyphenylacetaldehyde (DOPAL) and pyrogallol and glyoxal or methylglyoxal, because of the non-volatile nature of the latter compounds. Based on electrophilic aromatic substitution proposed in the literature,^23,42^ we searched for putative chemical structures of adducts as outlined in Fig. 3. According to the experimental conditions in the condensed e-liquid, not all the compounds envisaged were detected in mass spectrometry analyses as a consequence of low concentrations or as a result of intramolecular rearrangements leading to molecular structures different from those hypothesized. Working on precursor ions detection, we obtained structural information on molecular ion starting with selected ion monitoring mode (SIM). Table 1 shows the main putative adducts identified in this study, along with corresponding retention times, precursor and product ions. For the experiment with gallic acid, a mechanism of decarboxylation with the formation of pyrogallol has been hypothesized for its high scavenging activity of dicarbonyls.^24^Fig. 4 illustrates the abundance of each adduct in the model system investigated upon MRM experiments.

**Table 1:** Mass spectrometric data of the adducts hypothesized and identified in condensed e-liquid samples in which the water fraction was replaced by a solution containing gallic acid, hydroxytyrosol or epigallocatechin gallate, at different concentrations. GO, glyoxal; MGO, methylglyoxal; Pyr, pyrogallol; DOPAL, 3,4-dihydroxyphenylacetaldehyde; n.d., not detected

Adducts	RT (min)	[M − H]^−^ (*m*/*z*)	Fragment ions (*m*/*z*)	Collision energy (V)
**Gallic acid (GA)**				
GA + GO	n.d.	n.d.	n.d.	—
GA + 2GO	n.d.	n.d.	n.d.	—
GA + MGO	15.11	241.20	209.25	15
GA + 2MGO	6.67	313.10	75.05, 91.05	43, 15
GA + GO + MGO	n.d.	n.d.	n.d.	—
Pyr + GO	n.d.	n.d.	n.d.	—
Pyr + 2GO	n.d.	n.d.	n.d.	—
Pyr + MGO	n.d.	n.d.	n.d.	—
Pyr +2MGO	n.d.	n.d.	n.d.	—
Pyr + GO + MGO	n.d.	n.d.	n.d.	—

**Hydroxytyrosol (HT)**				
DOPAL + GO	15.17	209.10	153.15, 79.00	14, 22
DOPAL + MGO	n.d.	n.d.	n.d.	—
HT + GO	n.d.	n.d.	n.d.	—
HT + MGO	n.d.	n.d.	n.d.	—

**Epigallocathechin gallate (EGCG)**				
EGCG + GO	n.d.	n.d.	n.d.	—
EGCG + 2GO	n.d.	n.d.	n.d.	—
EGCG + MGO	n.d.	n.d.	n.d.	—
EGCG + 2MGO	30.66	567.10	299.15	15
	31.16	567.10	299.15	15
EGCG + GO + MGO	n.d.	n.d.	n.d.	—

For gallic acid and hydroxytyrosol, we identified two, and one adduct, respectively. In particular, we ascertained the occurrence of mono- and di-methylglyoxal adducts of the hydroxybenzoic derivative. In the case of mono-methylglyoxal adducts deriving from the reaction of gallic acid with methylglyoxal, we observed a major peak with a molecular ion at *m*/*z* 241 [M − H]^−^ (Fig. 4), which generated a fragment ion at *m*/*z* 209 [M − H]^−^ compatible with parental molecular structure, resulting from the loss of the oxygen atoms present at two hydroxyl groups. Whereas the mono-methylglyoxal adduct with gallic acid was evident in both liquid and vaped samples, the di-methylglyoxal adduct was detected only in vaped samples (Fig. 4). The products deriving from the reaction of glyoxal with gallic acid were not observed. These findings may be associated with higher reactivity of methylglyoxal toward polyphenols with respect to glyoxal, as already described by previous studies.^48^

Regarding hydroxytyrosol, we did not identify its mono- and di-molecular adducts of glyoxal and methylglyoxal, but the mono-glyoxal adduct of a corresponding degradation product, namely 3,4-dihydroxyphenylacetaldehyde (DOPAL) (Table 1). Navarro and Morales^29^ investigated the mechanism of methylglyoxal trapping by hydroxytyrosol monitoring the degradation of HT and the formation of related compounds of degradation. They found that, after 168 h at 37 °C in physiological conditions mimicking biological fluids, the amount of HT decreased (>98.2%) linearly over the time of the incubation. Using HPLC-ESI-QTOF-MS technique, authors identified as HT degradation products two main molecules: DOPAC and DOPAL (3,4-dihydroxyphenyl-acetic acid and 3,4-dihydroxyphenyl-acetaldehyde, respectively). They assume that HT oxidizes to DOPAL and later to DOPAC, which undergo through electrophilic aromatic substitution with methylglyoxal. Since no adducts of HT with methylglyoxal and glyoxal were detected, we envisaged a preference of the hydroxytyrosol degradation product for glyoxal, presumably as a result of the steric hindrance of the methyl group of methylglyoxal and different reaction kinetics of these dicarbonyl compounds. The different reaction conditions (temperature, time and solvent) could explain why we only found DOPAL as degradation product, but further investigations are needed to better understand the mechanism of reaction.

**Fig. 5:**
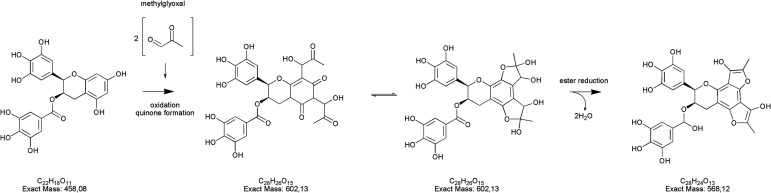
Putative pathway leading to the formation of the epigallocatechin gallate adduct (*m*/*z* [M − H]^−^ 567) as degradation product of the EGCG plus two methylglyoxal residues.

Regarding epigallocatechin gallate derivatives, we identified a putative precursor ion at *m*/*z* [M − H]^−^ 567 that suggested a preliminary adduction with two methylglyoxal molecules (C_28_H_26_C_15_, exact mass 602.13), followed by oxidation into quinone and finally an intramolecular aldol condensation yielding the formation of two additional rings. The final dehydration of two hydroxyl groups introduced two molecular unsaturation. In the presence of reducing agents in combination with a high concentration of glycol, we postulated a reduction of the benzoate ring into alcohol upon complexation of cation, according to the procedure detailed by Santaniello and co-workers that investigated the reduction of several esters into alcohol by means of sodium borohydride and polyethylene glycols.^49^ The mechanism reported in Fig. 5 is putatively and essentially based on Cannizzaro reaction^50^ with a preliminary oxidation of the hydroxyl group into carbonyl and the consequent reaction with methyl group, finally the removal of the two water molecules yielded the structure depicted in Fig. 5 with a [M − H]^−^ signal at *m*/*z* 567 (C_28_H_24_C_13_, exact mass 568.12). This precursor ion generated a main fragment at *m*/*z* 299 [M − 268 − H]^−^, which is fully compatible with the chemical structure hypothesized. In this respect, the high temperature reached during vaping played a crucial role in propylene glycol and glycerol transformation, as different adducts were identified in final reaction products with respect to those ascertained in previous studies.^23^ Thus, it is possible that under the experimental conditions, which involves a rapid increase in temperature, the di-methylglyoxal adduct of epigallocatechin gallate already described by Sang *et al.*^23^ for the reaction performed about 37 °C further decomposes to yield different molecular species with a chemical structure and an exact mass similar to the one postulated in Fig. 5. An alternative mechanism to the one reported in Fig. 5 is based on the condensation of carbonyl group with a final formation of two pyran rings, which also yielded a final product with a [M − H]^−^ signal at *m*/*z* 567. Further studies based on the use of isotopically-labelled reagents and additional spectroscopic/spectrometric techniques, as well as UV characterization are necessary to finally address this issue.

Relevant components of our model e-liquids (propylene glycol, glycerol and water) degraded prior to vaping, and the addition of polyphenols led to the formation of adducts with dicarbonyls. The same adducts were also identified in the vaped condensate. This suggests that dicarbonyls have formed in the e-liquids prior to vaping, presumably as degradation products of the major ingredients. Bekki *et al.*^51^ attributed the formation of carbonyls and dicarbonyls in e-cigarette aerosols to the oxidation of the e-liquid, when it becomes in contact with the heated atomiser coil in the presence of atmospheric oxygen. The present study suggests that the compounds observed by Ooi *et al.*^52^ are formed in the e-liquid of our model system prior to vaping, in part or in the whole. The difficulty in quantifying the concentration of these adducts prevented a fuller assessment of the role of each phenol compounds in reducing carbonyl formation. Nevertheless, it seems that the addition of appropriate polyphenols to the e-liquid potentially limited the production of toxic carbonyls, whether prior to vaping, during vaping-related heating, and/or in the aerosol. Along with glycerol and propylene glycol, other additives deserve further attention: Wu and O'Shea showed that the vaping of the viscous lipid oil vitamin E acetate has the potential to produce phenyl acetate and the toxic ketene, which may be a contributing factor to pulmonary injuries associated with using e-cigarette/vaping products.^53^ In this respect, pyrolysis experiments and study of degradation of polyphenols can open new scenario on the formation chemical routes behind the trapping of carbonyls compounds.

Regarding the nature of the toxic molecules formed in e-liquids, the degradation of glycerol was already suggested to play a central role in the formation of intermediates as hydroxypropanal, acetol and 2,3-hydroxypropanal, with the latter compound being a key precursor in the formation of methylglyoxal and 2,3-butanedione.^54^ In particular, methylglyoxal is formed in large amounts during catalytic conversion of glycerol *via* 2,3-hydroxypropanal; conversely, acetol generates only trace amounts of reactive α-dicarbonyls.^55^ The nature of the compounds here ascertained provided information on the most reactive dicarbonyl molecules present in e-liquids.

### Cytotoxicity of liquid and aerosol samples on two lung cell lines

**Fig. 6:**
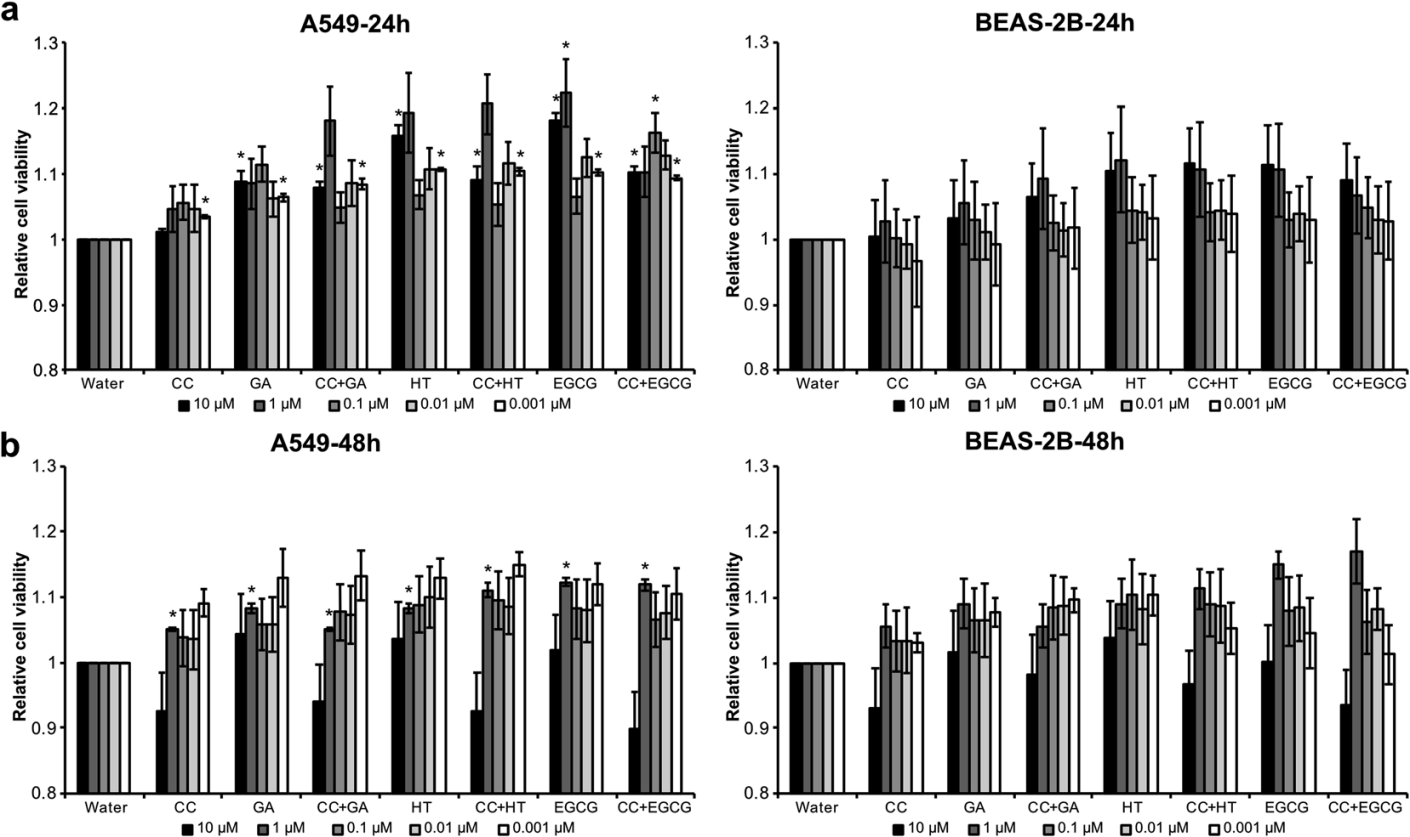
Short-term cytotoxic effect of e-cigarette aerosols on two lung cellular models, alveolar (A549) and bronchial (BEAS-2B) cell lines. Data were subjected to one-way ANOVA and significant differences (*P* ≤ 0.05) between means were determined using Tukey's test. Asterisks indicate differences between treatments of the same dilution in relation to the control water. Bars are the mean value of three replicates ± SE, *n* = 3. CC, condensed control; EGCG, epigallocatechin gallate; GA, gallic acid; HT, hydroxytyrosol.

The short-term cytotoxic effects of e-cigarette aerosols on two lung cellular models, namely alveolar (A549) and bronchial (BEAS-2B) cell lines, was also investigated (Fig. 6). A series of dilutions of the e-cigarette aerosols were used to examine their cytotoxicity relative to vehicle control. Moreover, we examined whether the RCS-trapping phenolics, gallic acid, hydroxytyrosol and epigallocatechin gallate, can attenuate the cytotoxicity of e-cigarettes. In addition to condensed aerosols, we also tested the cytotoxicity of the polyphenols that were previously diluted in water. Regardless of cell line and incubation period, the viability of both alveolar A549 and bronchial BEAS-2B cells was overall similar between treated and control samples. We apparently observed some discernible and slight increase in cell viability for both cell lines when the e-cigarette liquid was combined with the polyphenols, especially at 10, 1, and 0.01 μM concentrations after 24 h. This increased viability was slightly higher for the alveolar A549 cells than for the BEAS-2B cells, but overall, the two cell lines responded similarly to the treatments. These findings suggest that the formation of adducts does not have a negative impact on the cell viability within a 24–48 h period of treatment.

In conclusion, our work has established the ability of polyphenols to trap RCS in laboratory-formulated liquids vaped at high power (30 W), suggesting their potential value in commercial e-liquid formulations for reducing the levels of harmful carbonyls to which vapors are exposed. Overall, this study identifies a potential public health benefit in the apparently inhibiting effect of polyphenols on carbonyl production in e-cigarette liquids and emissions. The magnitude of this inhibiting effect, particularly its application to real-world vaping using other formulations and vaped at other power settings, and its potential impact on vaping populations require further dedicated investigations. This study also provides original information on the potential toxic activity of the resulting dicarbonyl-polyphenol adducts, revealing that the newly generated compounds in e-cig aerosols have no effect on cell viability. However, further studies are required in order to establish a cause–effects relationship between polyphenols and RCS.

## Funding source

The Carnegie Trust for the Universities of Scotland is thanked for providing funding to support this research (Grant Reference 50408).

## Conflicts of interest

Authors Bruna de Falco, W. Edryd Stephens and Alberto Fiore are associated with a patent pending on content relating to this manuscript.

## Supplementary Material
